# Double burden of malnutrition among women in reproductive Age (15–49 years) in Sierra Leone: a secondary data analysis of the demographic health survey 2019 (SLDHS-2019)

**DOI:** 10.1186/s40795-023-00795-w

**Published:** 2023-11-20

**Authors:** Eric Nzirakaindi Ikoona, Mame Awa Toure, Amon Njenga, Lucy Namulemo, Ronald Kaluya, Kassim Kamara, Freddy Wathum Drinkwater Oyat, Judith Aloyo, John Bosco Matovu, David Lagoro Kitara

**Affiliations:** 1 ICAP at Columbia University, Freetown, Sierra Leone; 2 Foothills Community Based interventions, Monticello, Columbia, KY USA; 3 Lindsey Wilson College, School of Professional Counseling, Columbia, KY USA; 4 Uganda Counseling and Support Services, Kampala, Uganda; 5 Directorate of Health Security and Emergencies, Ministry of Health and Sanitation, Freetown, Sierra Leone; 6 Uganda Medical Association (UMA), UMA-Acholi Branch, Gulu City, Uganda; 7 Rhites-N, Acholi, Gulu City, Uganda; 8 ICAP at Columbia University, Nairobi, Kenya; 9 Gulu Centre for Advanced Medical Diagnostics, Research, Trainings, and Innovations (GRUDI BIONTECH INITIATIVE), Gulu, Uganda; 10 Faculty of Medicine, Department of Surgery, Gulu University, P.0. Box 166, Gulu City, Uganda

**Keywords:** Malnutrition, Double burden of Malnutrition, Women, Sierra Leone, DHS of 2019

## Abstract

**Background:**

The double burden of malnutrition (DBM) is rising globally, particularly in sub-Saharan Africa. In Sierra Leone, the incidence of overweight, obesity (OWOB), and overnutrition among women has sharply increased. This finding accompanies the high incidence of undernutrition, which has been prevalent for decades. This study aimed to determine the prevalence of different malnutrition categories (underweight, overweight, obesity, and overnutrition) and associated factors among women of reproductive age (15–49 years) in Sierra Leone using secondary data analysis of the Sierra Leone Demographic Health Survey of 2019 (SLDHS-2019).

**Methods:**

We conducted secondary data analysis of the SLDHS-2019 of 7,514 women aged 15–49 years. We excluded pregnant, post-natal, lactating, and post-menopausal women. Data was collected using validated questionnaires, and respondents were selected through a multistage stratified sampling approach. A multivariable logistic regression analysis was used to determine factors associated with malnutrition among 15–49-year-old women in Sierra Leone.

**Results:**

Among 15–49-year-old women in Sierra Leone, the prevalence of underweight was 6.7% (95%CI: 4.5-8.9%); overweight at 19.7% (95%CI: 17.7-21.7%); obesity was 7.4% (95% CI: 5.2-9.6%); and overnutrition, 27.1% (95%CI: 25.2-29.0%). Women aged 25–34 years were more likely to be underweight (adjusted Odds Ratios, aOR = 1.670, 95%CI: 1.254–2.224; *p* < 0.001) than those aged 15–24 years; women who were not married were less likely to be underweight (aOR = 0.594, 95%CI: 0.467–0.755; *p* < 0.001) than married women. Women from the North were less likely to be underweight (aOR = 0.734, 95%CI: 0.559–0.963; *p* = 0.026) than the East, and those who did not listen to the radio were less likely to be underweight (aOR = 0.673; 95%CI: 0.549–0.826; *p* < 0.001) than those who did. Overweight was less likely among 25–34 years (aOR = 0.609, 95%CI: 0.514–0.722; *p* < 0.001) and 35–49 years (aOR = 0.480, 95%CI: 0.403–0.571; *p* < 0.001) age-groups than 15–24 years; more likely among not married women (aOR = 1.470, 95%CI:1.249–1.730; *p* < 0.001) than married; less likely among working-class (aOR = 0.840, 95%CI: 0.720–0.980; *p* = 0.026) than not working-class; most likely in women from the North (aOR = 1.325, 95%CI:1.096–1.602; *p* = 0.004), and less likely among women from the South (aOR = 0.755, 95%CI: 0.631–0.903; *p* = 0.002) than the East; less likely among women of middle-wealth-index (aOR = 0.656, 95%CI: 0.535–0.804; *p* < 0.001), richer-wealth-index (aOR = 0.400, 95%CI: 0.309–0.517; *p* < 0.001), and richest-wealth-index (aOR = 0.317, 95%CI: 0.234–0.431; *p* < 0.001) than the poorest-wealth-index; and more likely among women who did not listen to radios (aOR = 1.149; 95%CI:1.002–1.317; *p* = 0.047) than those who did. The predictors of overweight among women 15–49 years are the same as obesity and overnutrition, except overnutrition and obesity were less likely in female-headed households (aOR = 0.717,95%CI: 0.578–0.889; *p* < 0.001).

**Conclusion:**

The prevalence of all categories of malnutrition among women of reproductive age in Sierra Leone is high, affirming a double burden of malnutrition in this study population. Underweight was more likely among the 25–34-year age group than 15–24-year. The predictors of overweight, obesity, and overnutrition were being unmarried/single, residing in the North, and not listening to the radio. There is an urgent need for policymakers in Sierra Leone to design comprehensive educational programs for women of reproductive age on healthy lifestyles and the dangers of being underweight or over-nourished.

## Introduction

Malnutrition, in all its different forms, affects all countries worldwide [1]. Many countries face a double burden of malnutrition (DBM), where undernutrition and overweight and obesity (OWOB) exist in the same population, communities, households, and even individuals [2]. While OWOB is usually more prevalent in high-income countries among people with a low socioeconomic status (SES), the opposite has been observed in low-income countries [3, 4]. This increase in the prevalence of the DBM has also been observed in Asia [2]. Most undernourished people in Africa are in the sub-Saharan region, and hunger has risen since 2014 [5]. As of 2019, the number of undernourished people has been significant in Africa’s Eastern and middle subregions, reaching 27% and 29% of the total population, respectively [6].

Undernutrition and micronutrient deficiencies are, however, not the only nutrition concerns globally [7]. In 2016, 24% of all overweight children under five worldwide were also from Africa, with rates increasing among adolescents and young women [7].

In 2015, approximately 9% of the world’s adult population was underweight, and 30–40% were overweight or obese [8–10]. Women had a slightly higher prevalence of overweight and obesity than males [8, 9]. Although there is a marginal decline in the proportion of underweight [8, 9], the rise in the proportion of people being overweight or obese is a global pandemic [8–10].

There has been a 50–80% increase in overweight and obesity in the last 30 years [9, 10]. Despite variabilities in findings, the prevalence of OWOB has increased in most countries, ages, socioeconomic levels, and in both sexes [8–10]. In addition, being overweight and obese affects the functioning and structures of our body organs, thus increasing the risk of mortality [11, 12]. An abnormal nutritional status is one of the leading risk factors for premature death and loss of disability-adjusted life years (DALYs) [13].

Overweight and obesity are associated with many adverse health outcomes, including type 2 diabetes mellitus, cardiovascular diseases, some types of cancers, musculoskeletal and mental health disorders, and pregnancy-related complications [11, 14, 15]. Amongst many others, undernutrition increases the risks of infections [16], pre-term births, and low birth weights of offspring [15, 17].

Also, results from a recent analysis including 126 low-and-middle-income countries (LMIC) showed that countries with low Gross Domestic Products (GDP) drove the increase in the global DBM, as they had a more significant rise in OWOB and a slight decrease in the prevalence of undernutrition [3, 4, 10].

The state of malnutrition in sub-Saharan Africa epitomizes the DBM, with a high prevalence of undernutrition and increasing overnutrition (overweight and obesity), and both of these conditions contribute to diet-related noncommunicable diseases (NCDs) [7].

Notably, experts have argued that it is not likely that the sub-Saharan Africa region will achieve the Sustainable Development Goals (SDGs) of ending hunger and all forms of malnutrition by 2030 if these prevalence rates are maintained [18].

Increasingly, sub-Saharan Africa is now experiencing a DBM with high levels of undernutrition and a growing burden of overweight, obesity, and diet related NCDs [7]. The prevalence of undernutrition has substantially increased in sub-Saharan Africa between 2010 and 2016 [7].

Although chronic undernutrition is decreasing in Africa, children under five are increasingly stunted due to infections, hunger, and rapid population growth [7]. For now, overweight and obesity are increasing in all age groups, with girls and women being more affected than boys and men [7].

Interestingly, the drivers of the DBM are known to originate from outside the health sector (for example, poverty, hunger, and diseases are the main drivers of malnutrition in the African region and are linked with poor living conditions, lack of education, insecure livelihoods, and lack of access to essential services including healthcare and healthy life, safe, and nutritious foods), and operate across national and regional boundaries [7].

A study by Alaba et al. 2023, in ten sub-Saharan African countries, including Sierra Leone, found that the DBM was more prevalent among children under five years and that the poor suffered more from the DBM than the wealthy. For example, in Sierra Leone, children in the poorest socio-economic status (SES) had a prevalence of 32.5% and the richest SES quintile,12.1% [19]. Also, in the same study, the prevalence of DBM among children under five years was highest in Burundi at 27.4%, 14.3% in Sierra Leone, and least in Senegal at 8.6% [19].

Therefore, the increasing incidence and prevalence of DBM in many African countries drove this research team to investigate the factors associated with different categories of malnutrition among women in the reproductive age (15–49 years) in Sierra Leone using secondary data analysis of the 2019 Demographic Health Survey (SLDHS-2019).

The research team intends to present findings of this study to policymakers in Sierra Leone to plan and strategize on how to mitigate the effects of the DBM on the population of Sierra Leone.

## Methods

### Study design

We conducted a secondary data analysis of the SLDHS-2019 datasets.

### Data collection

This data was collected from May 14, 2019, to August 31, 2019 [20]. It was a nationally representative survey carried out by the Bureau of Statistics Sierra Leone as part of the international MEASURE demographic health surveys (DHS) with the support of Inner-City Fund (ICF) International and the United States Agency for International Development (USAID) [20]. SLDHS is a periodic survey conducted every five years in Sierra Leone, and the 2019 survey was the third, with the second completed in 2014 and the first in 2010 [20]. Five validated questionnaires based on the DHS program’s standard demographic and health survey (DHS-7) T4 were adapted to reflect the population and health issues relevant to Sierra Leone and used for the SLDHS-2019 [20]. These questionnaires include the Household Questionnaire (HQ), the Woman’s Questionnaire (WQ), the Man’s Questionnaire (MQ), the Biomarker Questionnaire (BMQ), and the Fieldworker Questionnaire (FWQ) [20]. The Sierra Leone Ethics and Scientific Review Committee (SLESRC) and the ICF Institutional Review Board reviewed and approved the survey protocol [20]. All questionnaires were in English, and the SLDHS-2019 used computer-assisted personal interviewing (CAPI) for data collection [20].

Household questionnaires collected data on household members, environment, assets, and basic demographic information. In contrast, women’s questionnaires collected data on women’s background characteristics, reproductive health, domestic violence, and nutritional status [20].

Regarding anthropometric measurements, weight was recorded in kilograms (kg) to the nearest decimal point and was measured using an electronic scale (SECA 878) [20]. Respondents’ height was measured using a stadiometer in centimeters (cm) to one decimal point [20].

### Study settings

As of July 2019, Sierra Leone had a population of 8.2 million people, with a total land area of 78,000 km^2^, with 23.8% of the population residing in urban areas [21]. Sierra Leone’s health system has six levels, from the highest level at national referral hospitals to the lowest level at the community [22]. Agriculture contributes about 24% of GDP, providing half of the export earnings and a primary source of income for 84% of Sierra Leone living in rural areas [23].

### Sampling and study participants

The 2015 population and housing census of the Republic of Sierra Leone conducted by Statistics Sierra Leone (Stats SL) provided the ready-made sampling frame for the SLDHS-2019 [23]. Sierra Leone is administratively divided into provinces and districts [21–23]. Each district is subdivided into chiefdoms/census wards, and each chiefdom/census ward is subdivided into Sects [21–23]. The 2015 population and housing census subdivided each locality into convenient census, Enumeration Areas (EAs) [23, 24]. The 2015 census EAs were the primary sampling units (PSUs) and clusters for the SLDHS-2019 [20–25]. The list of EAs from the 2015 census formed the basis for estimating the number of households and classified EAs (clusters) into urban or rural for the SLDHS-2019 sampling frame [20, 21, 23, 25].

The SLDHS-2019 employed a two-stage stratified sample design [20]. Stratification was achieved by separating each district into urban and rural areas [20, 25]. Thirty-one sampling strata were created, and samples were selected independently in each stratum via a two-stage selection process [20, 25]. Implicit stratifications were achieved at each lower administrative level by sorting the sampling frame before sample selection according to administrative order and using a probability proportional-to-size selection during the first sampling stage [20, 25]. In the first stage, five hundred and seventy-eight (578) EAs were selected with a probability proportional to EA size [20, 25]. In addition, the Enumeration Area (EA) size was determined by the number of households residing in it [20, 25]. A household listing operation was then performed in all selected EAs [20, 25]. The resulting lists of households served as a sampling frame for selecting households in the second stage of the survey [20, 25].

In the second stage’s selection, a fixed number of twenty-four households was chosen in every cluster through an equal probability systematic sampling, resulting in a total sample size of approximately 13,872 households distributed in 578 clusters [20, 25]. The household listing in this stage was conducted using computer tablets, and households were randomly selected through computer programming [20, 25].

The surveyors interviewed only the pre-selected households in the clusters, and no replacements or changes of the pre-selected households were allowed in the implementing stage of the survey to prevent selection bias in the study population [20, 25]. Due to the non-proportional allocation of samples to the different districts and the possible differences in response rates, sample weights were calculated, added to the data file, and applied so that the results would be representative at national and domain levels [20, 25]. Because the SLDHS-2019 sample was a two-stage stratified cluster sampling, sample weights were calculated separately at each sampling stage based on sampling probabilities [20, 25]. Thereafter, the SLDHS-2019 included all women aged 15–49 in the sample households [20, 25].

Permanent residents in the selected homes and visitors who stayed overnight before the survey were eligible for interviews in the household [20, 25]. The man’s questionnaire covered the identification of respondents, background information, reproduction, contraception, marriage and sexual activity, fertility preferences, employment status, gender roles, HIV and AIDS, and other health issues [25]. The biomarker questionnaire covered the identification of respondents, weights, heights, and hemoglobin measurements for children aged 0–5 years, weights, heights, HIV testing, and hemoglobin measurements for women aged 15–49 years [25].

The fieldworker questionnaire covered the background information on each field worker [25].

On the anthropometric measurements, weight was recorded in kilograms (kg) to the nearest decimal point and was measured using an electronic scale (SECA 878) [20, 25]. Participants’ heights were measured using a stadiometer in centimeters (cm) to one decimal point [20, 25]. The Body Mass Index (BMI) of individual women was calculated in kg/m^2^ using weights (in kilograms) and heights (meters) of women of reproductive age (15–49 years) and classified according to WHO criteria as underweight (< 18.5 kg/m^2^), normal weight (18.5–24.9 kg/m^2^), overweight (25.0–29.9 kg/m^2^), obese (≥ 30.0 kg/m^2^ and ≤ 50.0 kg/m^2^), and overnutrition (≥ 25.0 kg/m^2^ and ≤ 50.0 kg/m^2^).

To calculate each household’s wealth, we used wealth index (WI) as a proxy indicator of household wealth [25]. This composite index used household key asset ownership variables to calculate each household wealth index from the SLDHS-2019 data [25]. These variables were the characteristics of the household’s dwelling unit, for example, the source of water, type of toilet facilities, type of fuel used for cooking, number of rooms, ownership of livestock, possessions of durable goods, mosquito nets, and primary materials for the floor, roof, and walls of the dwelling place [25]. The respondent’s household wealth index was calculated using computer analysis of these household composite factors. It was then categorized into five quintiles as poorest, poorer, middle, richer, and richest wealth indices (Table 1).

**Table 1:** Sociodemographic characteristics of women of reproductive age (15–49 years) in Sierra Leone

Variable	Frequency (n = 7,514)	Percent (%)
**Ages (years)**		
15–24	2,916	38.8
25–34	2,176	29.0
35–49	2,422	32.2
**Parity**		
Never gave birth	1,895	25.2
Up to four	3,892	51.8
Five and above	1,727	23.0
**Residence**		
Urban	3,092	41.1
Rural	4,422	58.9
**Sex of the household head**		
Male	5,356	71.3
Female	2,158	28.7
**Household size**		
Less than six	2,995	39.9
Six and above	4,519	60.1
**Work status**		
Not working	2,280	30.3
Working	5,234	69.7
**Marital status**		
Married	4,795	63.8
Not married	2,719	36.2
**Regions of Sierra Leone**		
East	1,579	21.0
North	1,822	24.2
Northwest	1,026	13.7
South	1,831	24.4
Western	1,256	16.7
**Level of education attained**		
No formal educated	3,571	47.5
Primary	1,017	13.5
Secondary	2,641	35.2
Higher	285	3.8
**Wealth Index**		
Poorest	1,533	20.4
Poorer	1,428	19.0
Middle	1,531	20.4
Richer	1,634	21.7
Richest	1,388	18.5
**Watching TV**		
Yes	1,889	25.1
No	5,625	74.9
**BMI categories**		
Underweight (< 18.5 kg/m^2^)	502	6.7
Normal weight (18.5-24.99 kg/m^2^)	4,974	66.2
Overweight (25.0-29.99 kg/m^2^)	1,479	19.7
Obese (30.0-50.0 kg/m^2^)	559	7.4
Overnourished (≥ 25.0 kg/m^2^)	2,038	27.3
**Listening to radios**		
Yes	3,142	41.8
No	4,372	58.2
**Reading magazine**		
Yes	489	6.5
No	7.025	93.5
**Smoking**		
Yes	224	3.0
No	7,290	97.0
**Alcohol use**		
Yes	667	8.88
No	3,081	41.00

**The data source is SLDHS-2019.**

Table 1 shows that most Sierra Leone women of reproductive age were in the 15-24-year age group 2,916/7,514(38.8%); parity of up to four 3,892/7,514(51.8%); of rural residence 4,422/7,514(58.9%); male-headed households 5,356/7,514(71.3%); household size of six and above 4,519/7,514(60.1%); working-class 5,234/7,514(69.7%); married 4,795/7,514(63.8%); from the South 1,831/7,514(24.4%); normal BMI 4,974/7514(66.2%); had no formal education 3,571/7,514(47.5%); richer wealth index 1,634/7,514(21.7%); did not watch TV 5,625/7,514(74.9%); did not listen to radios 4,372/7,514(58.2%), did not read magazines 7,025/7,514(93.5%), did not smoke cigarettes 7,290/7,514(97.0%), and did not use alcohol 3,081/7,514(41.0%)

### Operational definitions

#### Body mass index (BMI)

Weight in kilograms divided by heights in meters squared (kg/m^2^).

#### Underweight

BMI < 18.5 kg/m^2^.

#### Overweight

BMI ≥ 25.0 kg/m^2^ and ≤ 29.9 kg/m^2^.

#### Obese

BMI ≥ 30.0 kg/m^2^ and ≤ 50.0 kg/m^2^.

#### Overnutrition (overweight and obese)

BMI ≥ 25.0 kg/m^2^ and ≤ 50.0 kg/m^2^.

#### Enumeration area (clusters)

An EA is a geographic area consisting of a convenient number of dwelling units that served as a counting unit for the survey.

### Statistical analysis

The SPSS analytic software version 24.0 complex samples package [25] was used for this analysis. Using a complex sample package accounted for the complex survey sampling while using sample weighted data accounted for the unequal probability sampling in different strata. Descriptive statistics and multivariable logistic regressions were used for data analysis. We used frequency tables and proportions/percentages to describe categorical variables, while means and standard deviations were used for continuous variables. Initially, we assessed each exposure variable separately for its association with the outcome variables (underweight, overweight, obesity, and overnutrition) using bivariate logistic regression, and we presented crude Odds Ratios (COR) at 95% Confidence Interval (CI) and *p*-values. Independent variables found insignificant at a *p*-value < 0.2 at bivariate analysis were added to the multivariable models [26–28]. Those variables with *p*-values > 0.201 at bivariate analysis were excluded from the multivariable analysis.

The multivariable logistic regression models included insignificant variables from bivariate analysis but were previously observed to be associated with underweight, overweight, obesity, and overnutrition in many studies. We constructed two multivariable logistic regression models by classifying independent variables into women’s individual, household, and community factors. We first performed a logistic regression model, which included only individual characteristics (age, parity, education level, working status, and marital status). After that, we constructed a final model that included individual characteristics adjusted for household and community characteristics (wealth index, residence, regions, household size, sex of household head, watching television, listening to radios, reading magazines, smoking, and alcohol use). The adjusted Odds Ratios (aOR) at 95% Confidence Intervals (CI) and *p*-values were calculated, with a statistical significance level set at *p*-value < 0.05 (Table 1).

### Sensitivity analysis

We conducted a sensitivity analysis with underweight women and those with normal BMI after excluding those with BMI above 25.0 kg/m^2^. The reported statistical values remain stable with no significant variations.

## Results

This study was conducted among women of reproductive age (15–49 years) in Sierra Leone, where lactating, postnatal, postmenopausal, and pregnant women in the age category were excluded from this population.

The socioeconomic characteristics of women in the reproductive age of 15–49 years (n = 7,514) were described by the Sierra Leone Demographic Health Survey of 2019 (SLDHS-2019) (Table 1).

### The prevalence of different malnutrition categories

The total female population aged 15–49 years who participated in the Demographic Health Survey of Sierra Leone (SLDHS-2019) was 15,574. The female population aged 15–49 with valid BMI results was 48%. Among women of reproductive age (15–49 years) with valid BMI measurements, 66.2% (95%CI:64.9-67.5%) had normal BMI.

The prevalence of different malnutrition categories among these women was as follows: underweight, 6.7%(95%CI:4.5-8.9%); overweight, 19.7%(95%CI:17.7-21.7%); obesity, 7.4%(95%CI:5.2-9.6%); and overnutrition 27.1%(95%CI:25.2-29.0%).

### Socio-economic and demographic characteristics of women in the reproductive age (15–49 years) in Sierra Leone

Most Sierra Leone women of reproductive age were in the 15–24 year age group (38.8%), parity of up to four (51.8%), of rural residence (58.9%), resided in male-headed households (71.3%), household size of six and above (60.1%), working-class (69.7%), married (63.8%), from the South (24.4%), had no formal education (47.5%), richer wealth index (21.7%), did not watch television (74.9%), did not listen to radios (58.2%), did not read magazines (93.5%), did not smoke cigarettes (97.0%), and did not use alcohol (41.0%) (Table 1).

### Categorization of women in the reproductive age in Sierra Leone by nutritional status

Of all the women that participated in this study, 502 (6.7%) were underweight; 4,974 (66.2%) had normal weight; 1,479 (19.7%) were overweight; 559 (7.4%) were obese, and 2,038 (27.3%) were overnourished. Underweight was commonest among the age group of 15–24 years (3.85%); normal weight among 15–24 years (28.44%), and overnutrition among the 35–49-year age group (11.54%) (Table 2).

**Table 2:** Descriptive statistics for the nutritional categories of respondents in the SLDHS-2019

	Underweight		Normal weight		Overnutrition	
Variables	Freq (n = 7,514)	(Percent) %	Freq (n = 7,514)	(Percent) %	Freq (n = 7,514)	(Percent) %
**Ages (years)**						
15–24	289	3.85	2,137	28.44	490	6.52
25–34	84	1.12	1,411	18.78	681	9.06
35–49	129	1.72	1,426	18.98	867	11.54
**Parity**						
Never gave birth	225	2.99	1,330	17.70	340	4.52
Up to four	182	2.42	2,537	33.76	1,173	15.61
Five and above	95	1.26	1,107	14.73	525	6.99
**Residence**		0.00		0.00		0.00
Urban	162	2.16	1,818	24.19	1112	14.80
Rural	340	4.52	3,156	42.00	926	12.32
**Sex of the household head**						
Male	343	4.56	3,621	48.19	1392	18.53
Female	159	2.12	1,353	18.01	646	8.60
**Household size**		0.00		0.00		0.00
Less than six	181	2.41	1,976	26.30	838	11.15
Six and above	321	4.27	2,998	39.90	1,200	15.97
**Work status**						
Not working	191	2.54	1,529	20.35	560	7.45
Working	311	4.14	3,445	45.85	1,478	19.67
**Marital status**		0.00		0.00		0.00
Married	232	3.09	3,102	41.28	1,461	19.44
Not Married	270	3.59	1,872	24.91	270	3.59
**Region**						
East	96	1.28	1,082	14.40	402	5.35
North	153	2.04	1,305	17.37	364	4.84
Northwest	73	0.97	724	9.64	229	3.05
South	134	1.78	1,173	15.61	524	6.97
Western	10	0.13	690	9.18	520	6.92
**Level of education**						
No formal education	211	2.81	2,399	31.93	961	12.79
Primary	96	1.28	686	9.13	235	3.13
Secondary	185	2.46	1,755	23.36	701	9.33
Higher	10	0.13	134	1.78	141	1.88
**Wealth index**						
Poorest	104	1.38	1,156	15.38	273	3.63
Poorer	120	1.60	1,053	14.01	255	3.39
Middle	121	1.61	1,050	13.97	360	4.79
Richer	97	1.29	974	12.96	563	7.49
Richest	60	0.80	741	9.86	587	7.81
**Watching TV**						
Yes	98	1.30	1,123	14.95	688	9.16
No	404	5.38	3,851	51.25	1370	18.23
**Listening to radios**						
Yes	152	2.02	1,967	26.18	1023	13.61
No	350	4.66	3,007	40.02	1015	13.51
**Reading of magazines**						
Yes	29	0.39	276	3.67	184	2.45
No	473	6.29	4,698	62.52	1854	24.67
**Smoking cigarettes**						
Yes	18	0.24	139	1.85	67	0.89
No	484	6.44	4,835	64.35	1971	26.23
**Alcohol use**						
Yes	35	0.47	429	5.71	203	2.70
No	140	1.86	2,005	26.68	936	12.46

Table 2 shows the nutritional classifications of women of reproductive age in Sierra Leone as per the SLDHS-2019. Underweight was commonest among the age group of 15–24 years, 289/7,514(3.85%); normal weight among 15–24 years, 2,137/7,514(28.44%) and overnutrition among the 35–49-year age group, 867/7,514(11.54%)

### BMI classifications of women of reproductive age (15–49 years) in Sierra Leone

The study found that all underweight women, 111(22.1%) were moderately thin and 159(31.7%) were mildly thin. The majority of women who were moderately thin (BMI = 16-17 kg/m^2^) were in the 20-29-year age group, 69(13.7%); mildly thin (BMI = 17.0-18.4 kg/m^2^) in the 40-49-year age group,72(14.3%); normal weight (BMI = 18.5-24.9 kg/m^2^) in the 20-29-year age group, 1173(23.6%); overweight (BMI = 25.0-29.9 kg/m^2^) in the 30–39-year age group, 411(27.8%); and obesity (BMI ≥ 30 kg/m^2^) in the 30–39-year age group, 188(33.6%) (Table 3).

**Table 3:** The BMI classification among women of reproductive age (15–49 years) in Sierra Leone (SLDHS-2019)

Variables	Moderately thin (BMI 16–17) (n = 502)	Mildly thin (BMI 17.0-18.4) (n = 502)	Normal weight (BMI = 18.5–24.9) (n = 4,974)	Overweight (BMI = 25.0-29.9) (n = 1,479)	Obese (BMI ≥ 30) (n = 559)
**Ages (years)**					
15–19	4(0.8)	18(3.59)	207(4.16)	26(1.76)	8(1.43)
20–29	69(13.7)	0(0.00)	1,173(23.58)	334(22.58)	92(16.46)
30–39	20(3.98)	69(13.7)	1,099(22.09)	411(27.79)	188(33.63)
40–49	18(3.59)	72(14.34)	738(14.84)	290(19.61)	169(30.23)
**Parity**					
Never gave birth	41(8.17)	169(33.67)	1,330 (26.74)	271(18.32)	69(12.34)
Up to four	34(6.77)	134(26.69)	2,537 (51.01)	844(57.07)	329(58.86)
Five and above	18(3.59)	71(14.14)	1,107 (22.26)	364(24.61)	161(28.80)
**Residence**					
Urban	10(1.99)	51(10.16)	960(19.30)	519(35.09)	282(50.45)
Rural	40(7.97)	160(31.87)	2,257(45.38)	542(36.65)	175(31.31)
**The sex of household-head**					
Male	35(6.97)	149(29.68)	2,422(48.69)	734(49.63)	288(51.52)
Female	15(2.99)	62(12.36)	581(11.68)	327(22.11)	169(30.23)
**Household size**					
Less than six	25(4.99)	80(15.94)	1,368(27.50)	467(31.58)	179(32.02)
Six and above	25(4.99)	131(26.10)	1,849(37.17)	594(40.16)	278(49.73)
**Work status**					
Not working	7(1.39)	39(7.77)	583(11.72)	206(13.93)	103(18.43)
Working	43(8.57)	172(34.24)	2,634(52.96)	855(57.89)	354(63.33)
**Marital status**					
Married	41(8.17)	169(36.67)	2,566(51.59)	858(58.01)	356(63.68)
Not married	9(1.79)	42(8.37)	651(13.09)	203(13.73)	101(18.07)
**Regions of Sierra Leone**					
East	10(1.99)	50(9.97)	702(14.11)	220(14.87)	88(15.74)
North	12(2.39)	48(9.56)	873(17.55)	203(13.73)	67(11.99)
Northwest	6(1.20)	37(7.37)	480(9.66)	122(8.25)	49(8.77)
South	16(3.19)	63(12.55)	782(15.72)	290(19.61)	115(20.57)
Western	6(1.20)	13(2.59)	380(7.64)	226(15.28)	138(24.69)
**Level of education**					
No formal education	33(6.57)	129(25.70)	1,983(39.87)	538(36.38)	235(42.04)
Primary	7(1.39)	25(4.99)	412(8.28)	136(9.20)	47(8.41)
Secondary	9(1.79)	52(10.36)	740(14.88)	288(19.47)	125(22.36)
Higher	2(0.40)	5(9.62)	82(1.65)	54(3.65)	50(8.94)
**Wealth index**					
Poorest	8(1.59)	59(11.75)	839(16.87)	180(12.18)	39(6.98)
Poorer	16(3.19)	64(12.75)	767(15.42)	43(2.99)	43(7.69)
Middle	17(3.39)	38(5.57)	720(14.48)	68(4.60)	68(12.15)
Richer	30(5.98)	30(5.38)	528(10.62)	141(9.53)	141(25.22)
Richest	1(0.20)	20(3.98)	363(7.30)	166(11.22)	166(29.70)
**Watching TV**					
Yes	5(1.59)	31(6.18)	603(12.12)	304(20.55)	171(30.60)
No	45(8.96)	180(35.86)	2,614(52.55)	757(51.18)	286(51.16)
**Listening to radio**					
Yes	12(2.39)	70(13.94)	1,210(24.33)	501(33.87)	256(45.80)
No	38(7.57)	141(20.09)	2,007(40.35)	560(37.86)	201(35.96)
**Reading Magazine**					
Yes	3(0.60)	7(1.39)	132(2.65)	67(4.54)	46(8.23)
No	47(9.36)	204(40.64)	3,085(62.02)	994(67.21)	411(73.52)
**Smoking**					
Yes	2(0.40)	14(2.79)	121(2.43)	39(2.64)	16(2.86)
No	48(9.56)	197(39.24)	3,096(62.24)	1.022(69.10)	441(73.52)
**Alcohol use**					
Yes	3(0.80)	30(5.98)	370(7.44)	124(8.38)	44(7.87)
No	30(5.98)	99(19.72)	1,639(32.95)	556(37.59)	229(40.97)

**The data source is SLDHS-2019.**

Table 3 shows the majority of underweight women who were moderately thin (BMI 16-17 kg/m^2^) were in the age group of 20–29 years, 69(13.7%) and mildly thin (BMI = 17.0-18.4 kg/m^2^) in 40–49 years, 72(14.34%). Women with normal weight (BMI = 18.5-24.9 kg/m^2^) in 20-29-year age group, 1173(23.58%); overweight (BMI = 25.0-29.9 kg/m^2^) in 30–39-year age group 411(27.79%), in obese (BMI ≥ 30 kg/m^2^) 188(33.63%) among 30–39-year age group

### Predictors of underweight among women (15–49 years) in Sierra Leone

Underweight among Sierra Leone women was more likely among the age group of 25–34 years (aOR 1.670 95%CI:1.254–2.224; *p* < 0.001) than 15–24 years; less likely among the unmarried/single (aOR 0.594 95%CI: 0.467–0.755; *p* < 0.001) than married; less likely among women from the North (aOR 0.734 95%CI: 0.559–0.963; *p* = 0.026) than the East, and less likely among women who did not listen to radios (aOR 0.673 95%CI 0.549–0.826; *p* < 0.001) than those who did (Table 4).

**Table 4:** Prevalence and predictors of underweight among respondents (15–49 years) in Sierra Leone

Variables	Underweight (n = 502) (n, %)	Normal weight (n = 4,974) (n, %)	Unadjusted COR	95% CI	*p* value	Adjusted OR	95% CI	*p* value
**Ages (years)**								
15–24	289(11.9)	2,137(88.1)	**Reference**			**Reference**		
25–34	84(5.6)	1,411(94.4)	2.272	(1.765–2.923)	< 0.001	1.670	(1.254–2.224)	< 0.001
35–49	129(8.3)	1,426(91.7)	1.495	(1.202–1.859)	< 0.001	1.136	(0.870–1.483)	0.350
**Parity**								
Up to four	407(9.5)	3,867(90.5)	**Reference**					
Five and above	95(7.9)	1,107(92.1)	1.226	(0.971–1.548)	0.086			
**Residence**								
Urban	162(8.2)	1,818(91.8)	**Reference**					
Rural	340(9.7)	3,156(90.3)	0.827	(0.680–1.006)	0.057			
**Sex household head**								
Male	343(8.7)	3,621(91.3)	**Reference**			**Reference**		
Female	159(10.5)	1,353(89.5)	0.806	(0.661–0.983)	0.033	0.925	(0.750–1.141)	0.469
**Household size**								
Less than six	181(8.4)	1,976(91.6)	**Reference**					
Six and above	321(9.7)	2,998(90.3)	0.855	(0.707–1.035)	0.109			
**Work status**								
Not working	191(11.1)	1,529(88.90	**Reference**					
Working	311(8.3)	3,445(91.7)	1.384	(1.144–1.673)	0.001	1.082	(0.868–1.349)	0.481
**Marital status**								
Married	232(7.0)	3,102(93.0)	**Reference**					
Not married	270(12.6)	1,872(87.4)	0.519	(0.431–0.624)	< 0.001	0.594	(0.467–0.755)	< 0.001
**Regions of Sierra Leone**								
East	96(8.1)	1,082(91.9)	**Reference**			**Reference**		
North	153(10.5)	1,305(89.5)	0.757	(0.579–0.989)	0.041	0.734	(0.559–0.963)	0.026
Northwest	73(9.2)	724(90.8)	0.88	(0.640–1.210)	0.431	0.840	(0.609–1.160)	0.290
South	134(10.3)	1,173(89.7)	0.777	(0.590–1.022)	0.071	0.776	(0.588–1.204)	0.073
Western	46(6.2)	690(93.8)	1.331	(0.925–1.916)	0.777	1.385	(0.954–2.011)	0.087
**Level of education**								
No formal education	211(8.1)	2,399(91.9)	**Reference**					
Primary	96(12.3)	686(87.7)	0.628	(0.487–0.812)	< 0.001			
Secondary	185(9.5)	1,755(90.5)	0.834	(0.679–1.026)	0.086			
Higher	10(6.9)	134(93.1)	1.179	(0.611–2.275)	0.624			
**Wealth Index**								
Poorest	104(8.3)	1,156(91.7)	**Reference**					
Poorer	120(10.2)	1,053(89.8)	0.789	(0.599–1.040)	0.093			
Middle	121(10.3)	1,050(89.7)	0.781	(0.593–1.028)	0.078			
Richer	97(9.1)	974(90.9)	0.903	(0.676–1.207)	0.491			
Richest	60(7.5)	741(92.5)	1.111	(0.798–1.547)	0.533			
**Watching TV**								
Yes	98(8.0)	1,123(92.0)	**Reference**					
No	404(9.5)	3,851(90.5)	0.832	(0.661–1.047)	0.117			
**Listens to radio**								
Yes	152(7.2)	1,967(92.8)	**Reference**					
No	350(10.4)	3,007(89.6)	0.664	(0.544–0.810)	< 0.001	0.673	(0.549–0.826)	< 0.001
**Reading of magazines**								
Yes	29(9.5)	276(90.5)	**Reference**					
No	473(9.1)	4,698(90.9)	1.044	(0.704–1.548)	0.832			
**Smokes cigarettes**								
Yes	18(11.5)	139(88.5)	**Reference**					
No	484(9.1)	4,835(90.9)	1.294	(0.785–2.132)	0.313			
**Alcohol use**								
Yes	35(7.5)	429(92.5)	**Reference**					
No	140(6.7)	2,005(93.3)	1.168	(0.795–1.717)	0.428			

**The data source is SLDHS-2019.**

aOR: Adjusted Odds Ratios; CI: Confidence Interval; COR: Crude Odds Ratio; SLDHS: Sierra Leone Demographic Health Survey

In Table 4, underweight Sierra Leone women in the reproductive age (15–49 years) were more likely among the age group of 25–34 years (aOR 1.670; 95%CI:1.254–2.224; *p* < 0.001); less likely among married/single women (aOR 0.594; 95%CI: 0.467–0.755; *p* < 0.001), less likely among women from the North (aOR 0.734; 95%CI:0.559–0.963; *p* = 0.026), and less likely among women who did not listen to radios (aOR 0.673; 95%CI: 0.549–0.826; *p* < 0.001)

### Predictors of overweight among women (15–49 years) in Sierra Leone

Overweight among Sierra Leone women was less likely among the age group of 25–34 years (aOR 0.609, 95%CI: 0.514–0.722; *p* < 0.001) and 35-49-year (aOR 0.480, 95%CI: 0.403–0.571; *p* < 0.001) than in 15–24 years, respectively; more likely among unmarried/single women (aOR 1.470, 95%CI:1.249–1.730; *p* < 0.001) than married; less likely among working-class women (aOR 0.840, 95%CI: 0.720–0.980; *p* < 0.026) than not working; more likely among women from the North (aOR 1.325, 95%CI:1.096–1.602; *p* = 0.004) than the East; less likely among women from the South (aOR 0.755, 95%CI: 0.631–0.903; *p* = 0.002) than the East; less likely among women of middle wealth index (aOR 0.656, 95%CI: 0.535–0.804; *p* < 0.001), richer wealth index (aOR 0.400, 95%CI: 0.309–0.517; *p* < 0.001) and richest wealth index (aOR 0.317, 95%CI: 0.234–0.431; *p* < 0.001) than poorest wealth index, respectively, and was more likely in women who did not listen to radios (aOR 1.149, 95%CI:1.002–1.317; *p* < 0.047) than those who did (Table 5).

**Table 5:** The prevalence and predictors of overweight among women (15–49 years) in Sierra Leone

Variables	Overweight (n = 1,479) (n, %)	Normal weight (n = 4,974) (n, %)	Unadjusted COR	95% CI	*p* value	Adjusted OR	95% CI	*p* value
**Ages (years)**								
15–24	411(16.1)	2,137(83.9)	**Reference**			**Reference**		
25–34	503(26.3)	1,411(73.7)	0.540	(0.466–0.625)	< 0.001	0.609	(0.514–0.722)	< 0.001
35–49	565(28.4)	1,426(71.6)	0.485	(0.420–0.560)	< 0.001	0.480	(0.403–0.571)	< 0.001
**Parity**								
Up to four	1,115(22.4)	3,867(77.6)	**Reference**					
Five and above	364(24.7)	1,107(75.3)	0.765	(0.756–1.005)	0.058			
**Residence**								
Urban	753(29.3)	1,818(70.7)	**Reference**			**Reference**		
Rural	726(18.7)	3,156(81.3)	1.801	(1.601–2.024)	< 0.001	1.049	(0.851–1.292)	0.653
**Sex of the household head**								
Male	1,041(22.2)	3,621(77.7)	**Reference**			**Reference**		
Female	438(24.5)	1,353(75.5)	0.806	(0.661–0.983)	0.033	0.893	(0.776–1.029)	0.119
**Household size**								
Less than six	615(23.7)	1,976(76.3)	**Reference**					
Six and above	864(22.4)	2,998(77.6)	1.080	(0.960–1.215)	0.201			
**Work status**								
Not working	400(20.7)	1,529(79.3)	**Reference**			**Reference**		
Working	1,079(23.9)	3,445(76.1)	0.835	(0.734–0.951)	0.006	0.840	(0.720–0.980)	0.026
**Marital status**								
Married	1,058(25.4)	3,102(74.6)	**Reference**			**Reference**		
Not Married	421(18.4)	1,872(81.6)	1.517	(1.336–1.721)	< 0.001	1.470	(1.249–1.730)	< 0.001
**Region**								
East	297(21.5)	1,082(78.5)	**Reference**			**Reference**		
North	278(17.6)	1,305(82.4)	1.289	(1.074–1.546)	0.006	1.325	(1.096–1.602)	0.004
Northwest	169(18.9)	724(81.1)	1.176	(0.952–1.452)	0.132	1.164	(0.936–1.449)	0.171
South	394(25.1)	1,173(74.9)	0.817	(0.688–0.970)	0.021	0.755	(0.631–0.903)	0.002
Western	341(33.1)	690(66.9)	0.555	(0.463–0.667)	< 0.001	0.922	(0.741–1.147)	0.465
**Level of education**								
No education	699(8.1)	2,399(77.4)	**Reference**					
Primary	180(12.3)	686(79.2)	1.110	(0.923–1.335)	0.266			
Secondary	521(9.5)	1,755(77.1)	0.981	(0.863–1.117)	0.777			
Higher	79(6.9)	134(62.9)	0.494	(0.370–0.661)	< 0.001			
**Wealth Index**								
Poorest	229(16.5)	1,156(83.5)	**Reference**			**Reference**		
Poorer	204(16.2)	1,053(83.8)	1.023	(0.832–1.257)	0.832	0.943	(0.763–1.165)	0.587
Middle	279(21.0)	1,050(79.0)	0.746	(0.614–0.905)	0.003	0.656	(0.535–0.804)	< 0.001
Richer	390(28.6)	974(71.4)	0.495	(0.412–0.595)	< 0.001	0.400	(0.309–0.517)	< 0.001
Richest	377(33.7)	741(66.3)	0.389	(0.323–0.470)	< 0.001	0.317	(0.234–0.431)	< 0.001
**Watching TV**								
Yes	454(28.8)	1,123(71.2)	**Reference**			**Reference**		
No	1,025(21.0)	3,851(79.0)	1.519	(1.335–1.728)	< 0.001	0.979	(0.821–1.168)	0.817
**Listens to radios**								
Yes	708(26.5)	1,967(73.5)	**Reference**			**Reference**		
No	771(20.4)	3,007(79.6)	1.404	(1.249–1.578)	< 0.001	1.149	(1.002–1.317)	0.047
**Reading of magazines**								
Yes	120(30.3)	276(69.7)	**Reference**			**Reference**		
No	1,359(22.4)	4,698(77.6)	1.503	(1.203–1.878)	< 0.001	1.188	(0.926–1.525)	0.176
**Smokes cigarettes**								
Yes	46(24.9)	139(75.1)	**Reference**					
No	1,433(22.9)	4,835(77.1)	1.117	(0.796–1.566)	0.523			
**Alcohol use**								
Yes	149(25.8)	429(74.2)	**Reference**					
No	686(25.5)	2,005(74.5)	1.015	(0.827–1.247)	0.886			

**The data source is SLDHS-2019**

In Table 5, overweight Sierra Leone women in the reproductive age were less likely among age group of 25–34 years (aOR 0.609; 95%CI: 0.514–0.722; *p* < 0.001); less likely among 35–49 years (aOR 0.480; 95%CI: 0.403–0.57; *p* < 0.001); more likely among the not married/single women (aOR 1.470; 95%CI:1.249–1.730; *p* < 0.001); less likely among working-class women (aOR 0.840; 95%CI: 0.720–0.980; *p* < 0.026); more likely among women from the North (aOR 1.325; 95%CI:1.096–1.602; *p* = 0.004); less likely among women from the South (aOR 0.755; 95%CI: 0.631–0.903; *p* = 0.002); less likely among women in the middle wealth index (aOR 0.656; 95%CI: 0.535–0.804; *p* < 0.001); less likely among richer wealth index (aOR 0.400; 95%CI: 0.309–0.517; *p* < 0.001); less likely among the richest wealth index (aOR 0.317; 95%CI: 0.234–0.431; *p* < 0.001), and more likely among those who did not listen to radios (aOR 1.149; 95%CI:1.002–1.317; *p* < 0.047)

### Predictors of obesity among women (15–49 years) in Sierra Leone

Table 6 shows that Sierra Leone women in the age group of 25–34-year (aOR 0.609, 95%CI: 0.514–0.722; *p* < 0.001) and 35-49-year (aOR 0.480, 95%CI: 0.403–0.571; *p* < 0.001) were less likely to be obese than the age group of 15-24-year, respectively. Obesity was less likely among female-headed households (aOR 0.717, 95%CI: 0.578–0.889; *p* < 0.001) than male-headed households; less likely among working-class women (aOR 0.840, 95%CI: 0.720–0.980; *p* < 0.026) than not working-class; more likely among women from the North (aOR 1.447, 95%CI:1.054–1.985; *p* = 0.022) and less likely among women from the South (aOR 0.740, 95%CI: 0.552–0.991; *p* = 0.043) than the East, respectively. Obesity was less likely among the middle wealth index (aOR 0.418, 95%CI: 0.283–0.618; *p* < 0.001), richer wealth index (aOR 0.156, 95%CI: 0.101–0.242; *p* < 0.001), and richest wealth index (aOR 0.095, 95%CI: 0.058–0.155; *p* < 0.001) compared to poorest wealth index, respectively. In addition, obesity was more likely among women who did not listen to radios (aOR 1.370, 95%CI:1.105–1.699; *p* < 0.004) than those who did.

**Table 6:** The prevalence and predictors of obesity among women (15–49 years) in Sierra Leone

Variables	Obese (n = 559) (n, %)	Normal weight (n = 4,974) (n, %)	Unadjusted COR	95% CI	*p* value	Adjusted OR	95% CI	*p* value
**Ages (years)**								
15–24	79(3.6)	2,137(96.4)	**Reference**			**Reference**		
25–34	178(11.2)	1,411(88.8)	0.293	(0.223–0.385)	< 0.001	0.265	(0.196–0.359)	< 0.001
35–49	302(17.5)	1,426(82.5)	0.175	(0.135–0.226)	< 0.001	0.122	(0.090–0.164)	< 0.001
**Parity**								
Up to four	398(9.3)	3,867(90.7)	**Reference**					
Five and above	161(12.7)	1,107(87.3)	0.708	(0.582–0.860)	0.001	1.243	(0.976–1.583)	0.078
**Residence**								
Urban	359(16.5)	1,818(83.5)	**Reference**			**Reference**		
Rural	200(6.0)	3,156(94.0)	3.116	(2.597–3.739)	< 0.001	0.977	(0.706–1.353)	0.889
Sex of the household head								
Male	351(351)	3,621(91.2)	**Reference**			**Reference**		
Female	208(208)	1,353(86.7)	0.631	(0.525–0.757)	< 0.001	0.717	(0.578–0.889)	0.002
**Household size**								
Less than six	223(10.1)	1,976(89.9)	**Reference**					
Six and above	336(10.1)	2,998(89.9)	1.007	(0.842–1.204)	0.939			
**Work status**								
Not working	160(9.5)	1,529(90.5)	**Reference**					
Working	399(10.4)	3,445(89.6)	0.904	(0.745–1.096)	0.303			
**Marital status**								
Married	403(11.5)	3,102(88.5)	**Reference**			**Reference**		
Not Married	156(7.7)	1,872(92.3)	1.559	(1.285–1.892)	< 0.001	1.293	(1.001–1.669)	< 0.049
**Region**								
East	104(8.8)	1,082(91.2)	**Reference**			**Reference**		
North	86(6.2)	1,305(93.8)	1.459	(1.084–1.963)	0.013	1.447	(1.054–1.985)	0.022
Northwest	60(7.7)	724(92.3)	1.160	(0.833–1.616)	0.381	1.107	(0.777–1.577)	0.573
South	130(10.0)	1,173(90.0)	0.867	(0.662–1.137)	0.303	0.740	(0.552–0.991)	0.043
Western	179(20.6)	690(79.4)	0.371	(0.286–0.480)	< 0.001	0.857	(0.628–1.169)	0.331
**Level of education**								
No formal education	262(9.8)	2,399(90.2)	**Reference**					
Primary	55(7.4)	686(92.6)	0.628	(0.487–0.812)	< 0.001			
Secondary	180(9.3)	1,755(99.7)	0.834	(0.679–1.026)	0.086			
Higher	62(31.6)	134(88.4)	1.179	(0.611–2.275)	0.624			
**Wealth Index**								
Poorest	44(3.7)	1,156(96.3)	**Reference**			**Reference**		
Poorer	51(4.6)	1,053(95.4)	0.786	(0.521–1.186)	0.251	0.705	(0.463–1.074)	0.104
Middle	81(7.2)	1,050(92.8)	0.493	(0.339–7.19)	< 0.001	0.418	(0.283–0.618)	< 0.001
Richer	173(15.1)	974(84.9)	0.214	(0.152–0.302)	< 0.001	0.156	(0.101–0.242)	< 0.001
Richest	210(22.1)	741(77.9)	0.134	(0.096–0.188)	< 0.001	0.095	(0.058–0.155)	< 0.001
**Watching TV**								
Yes	214(16.0)	1,123(84.0)	**Reference**			**Reference**		
No	345(8.2)	3,851(91.8)	2.127	(1.771–2.554)	< 0.001	0.880	(0.675–1.148)	0.347
**Listens to radio**								
Yes	315(13.8)	1,967(86.2)	**Reference**			**Reference**		
No	244(7.5)	3,007(92.5)	1.974	(1.654–2.355)	< 0.001	1.370	(1.105–1.699)	0.004
**Reading of magazines**								
Yes	64(18.8)	276(81.2)	**Reference**			**Reference**		
No	495(9.5)	4,698(90.5)	2.201	(1.651–2.933)	< 0.001	1.224	(0.869–1.723)	0.248
**Smokes cigarettes**								
Yes	21(13.1)	139(86.9)	**Reference**					
No	538(10.0)	4,835(90.0)	1.358	(0.851–2.167)	0.200			
**Alcohol use**								
Yes	54(11.2)	429(88.8)	**Reference**					
No	250(11.1)	2,005(88.9)	1.010	(0.739–1.379)	0.953			

**The data source is SLDHS-2019.**

In Table 6, obesity among Sierra Leone women in the reproductive age (15–49 years) was less likely in the age group of 25–34 years (aOR 0.609; 95%CI: 0.514–0.722; *p* < 0.001); less likely among 35–49 years (aOR 0.480; 95%CI: 0.403–0.57; *p* < 0.001); less likely among female-headed households (aOR 0.717; 95%CI: 0.578–0.889; *p* < 0.001); less likely among working-class women (aOR 0.840; 95%CI: 0.720–0.980; *p* < 0.026); more likely among women from the North (aOR 1.447; 95%CI:1.054–1.985; *p* = 0.022); less likely among women from the South (aOR 0.740; 95%CI: 0.552–0.991; *p* = 0.043), less likely among middle wealth index (aOR 0.418; 95%CI: 0.283–0.618; *p* < 0.001); less likely among the richer wealth index (aOR 0.156; 95%CI: 0.101–0.242; *p* < 0.001), less likely among the richest wealth Index (aOR 0.095; 95%CI: 0.058–0.155; *p* < 0.001); and more likely among women who did not listen to radios (aOR 1.370; 95%CI:1.105–1.699; *p* < 0.004)

### Predictors of overnutrition among women (15–49 years) in Sierra Leone

Table 7 shows that Sierra Leone women in the age group of 25–34 years (aOR 0.512; 95%CI: 0.438–0.599; *p* < 0.001) and 35-49-year age group (aOR 0.350, 95%CI: 0.298–0.41; *p* < 0.001) were less likely to have overnutrition than 15–24-year age-group, respectively. Overnutrition was less likely among female-headed households (aOR 0.836, 95%CI: 0.736–0.950; *p* < 0.006) than male-headed households; less likely among working-class women (aOR 0.840, 95%CI: 0.720–0.980; *p* < 0.026) than not working women; more likely among unmarried/single woman (aOR 1.432, 95%CI:1.235–1.660; *p* < 0.001) than married; more likely among women from the North (aOR 1.359, 95%CI:1.143–1.660; *p* = 0.001) and less likely among women from the South (aOR 0.750, 95%CI: 0.637–0.884; *p* = 0.001) than women from the East, respectively. In addition, overnutrition was less likely among the middle wealth index (aOR 0.603, 95%CI: 0.499–0.728; *p* < 0.001), richer wealth index (aOR 0.333, 95%CI: 0.264–0.42; *p* < 0.001), and the richest wealth index (aOR 0.248, 95%CI: 0.188–0.326; *p* < 0.001) than the poorest wealth index, respectively. However, it was more likely among women who did not listen to radios (aOR 1.201, 95%CI:1.061–1.359; *p* < 0.004) than those who did.

**Table 7:** Prevalence and predictors of overnutrition among women (15–49 years) in Sierra Leone

Variable	Overnutrition (n = 2,038) (n, %)	Normal weight (n = 4,974) (n, %)	Unadjusted COR	95% CI	*p* value	Adjusted OR	95% CI	*p* value
**Ages (years)**								
15–24	490(18.7)	2,137(81.3)	**Reference**			**Reference**		
25–34	681(32.6)	1,411(67.4)	0.475	(0.415–0.543)	< 0.001	0.512	(0.438–0.599)	< 0.001
35–49	867(37.8)	1,426(62.2)	0.377	(0.331–0.429)	< 0.001	0.350	(0.298–0.411)	< 0.001
**Parity**								
Up to four	1,513(28.1)	3,867(71.9)	**Reference**					
Five and above	525(32.2)	1,107(67.8)	0.825	(0.732–0.930)	0.002	1.151	(0.989–1.339)	0.070
**Residence**								
Urban	1,112(38.0)	1,818(62.0)	**Reference**			**Reference**		
Rural	926(22.7)	3,156(77.3)	2.085	(1.878–2.314)	< 0.001	1.050	(0.870–1.267)	0.611
**Sex of household head**								
Male	1,392(27.8)	3,621(72.2)	**Reference**			**Reference**		
Female	646(32.3)	1,353(67.7)	0.805	(0.720–0.901)	< 0.001	0.836	(0.736–0.950)	0.006
**Household size**								
Less than six	838(8.4)	1,976(70.2)	**Reference**					
Six and above	1,200(28.6)	2,998(71.4)	1.060	(0.954–1.177)	0.280			
**Work status**								
Not working	560(26.8)	1,529(73.2)	**Reference**			**Reference**		
Working	1,478(30.0)	3,445(70.0)	0.854	(0.761–0.957	0.007	0.886	(0.771–1.018)	0.088
**Marital status**								
Married	1,461(732)	3,102(68.0)	**Reference**			**Reference**		
Not married	270(12.6)	1,872(76.4)	1.528	(1.366–1.709)	< 0.001	1.432	(1.235–1.660)	< 0.001
**Region**								
East	401(27.0)	1,082(73.0)	**Reference**			**Reference**		
North	364(21.8)	1,305(78.2)	1.329	(1.129–1.564)	0.001	1.359	(1.143–1.616)	0.001
Northwest	229(24.0)	724(76.0)	1.172	(0.971–1.414)	0.098	1.151	(0.944–1.403)	0.164
South	524(30.9)	1,173(69.1)	0.830	(0.711–0.968)	0.017	0.750	(0.637–0.884)	0.001
Western	520(43.0)	690(57.0)	0.492	(0.418–0.578)	< 0.001	0.891	(0.733–1.083)	0.247
**Level of education**								
No formal education	961(28.6)	2,399(71.4)	**Reference**					
Primary	235(25.5)	686(74.5)	1.169	(0.991–1.380)	0.965			
Secondary	701(28.5)	1,755(71.5)	1.003	(0.894–1.125)	0.961			
Higher	141(651.3)	134(48.7	0.381	(0.297–0.880)	< 0.001			
**Wealth Index**								
Poorest	273(19.1)	1,156(80.9)	**Reference**			**Reference**		
Poorer	255(19.5)	1,053(80.5)	0.975	(0.806–1.179)	0.796	0.892	(0.733–1.086)	0.255
Middle	360(25.5)	1,050(74.5)	0.689	(0.576–0.823)	< 0.001	0.603	(0.499–0.728)	< 0.001
Richer	563(36.6)	974(63.4)	0.409	(0.345–4.83)	< 0.001	0.333	(0.264–0.421)	< 0.001
Richest	587(44.2)	741(55.8)	0.298	(0.251–0.354)	< 0.001	0.248	(0.188–0.326)	< 0.001
**Watching TV**								
Yes	688(37.3)	1,123(62.7)	**Reference**			**Reference**		
No	1,370(26.2)	3,851(73.8)	1.672	(1.492–1.874)	< 0.001	0.954	(813-1.118)	0.560
**Listens to radio**								
Yes	1,023(34.2)	1,967(65.8)	**Reference**			**Reference**		
No	1,015(25.2)	3,007(74.8)	1.541	(1.389–1.709)	< 0.001	1.201	(1.061–1.359)	0.004
**Reading magazine**								
Yes	184(40.0)	276(60.0)	**Reference**			**Reference**		
No	1,854(28.3)	4,698(71.7)	1.689	(1.391–2.051)	< 0.001	1.217	(0.974–1.521)	0.085
**Smokes cigarettes**								
Yes	67(32.5)	139(67.5)	**Reference**					
No	1,971(29.0)	4,835(71.0)	1.182	(0.879–1.590)	0.267			
**Alcohol use**								
Yes	203(32.1)	429(67.9)	**Reference**					
No	936(31.8)	2,005(68.2)	1.014	(0.843–1.219)	0.885			

In Table 7, overnutrition among Sierra Leone women (15–49 years) was less likely among the age group of 25–34 years (aOR 0.512; 95%CI: 0.438–0.599; *p* < 0.001); less likely among 35–49 years (aOR 0.350; 95%CI: 0.298–0.411; *p* < 0.001); less likely among female-headed households (aOR 0.836; 95%CI: 0.736–0.950; *p* < 0.006); less likely among working-class women (aOR 0.840; 95%CI: 0.720–0.980; *p* < 0.026); more likely among not married/single woman (aOR 1.432; 95%CI:1.235–1.660; *p* < 0.001); more likely among women from the North (aOR 1.359; 95%CI:1.143–1.660; *p* = 0.001); less likely among women from the South (aOR 0.750; 95%CI: 0.637–0.884; *p* = 0.001); less likely among middle wealth index (aOR 0.603; 95%CI: 0.499–0.728; *p* < 0.001); less likely among richer wealth index (aOR 0.333; 95%CI: 0.264–0.421; *p* < 0.001); less likely among women in the richest Wealth Index (aOR 0.248; 95%CI: 0.188–0.326; *p* < 0.001); and more likely among women who did not listen to radios (aOR 1.201; 95%CI:1.061–1.359; *p* < 0.004)

### The prevalence of underweight, overweight, obesity, and overnutrition by age group population in Sierra Leone

**Fig. 1:**
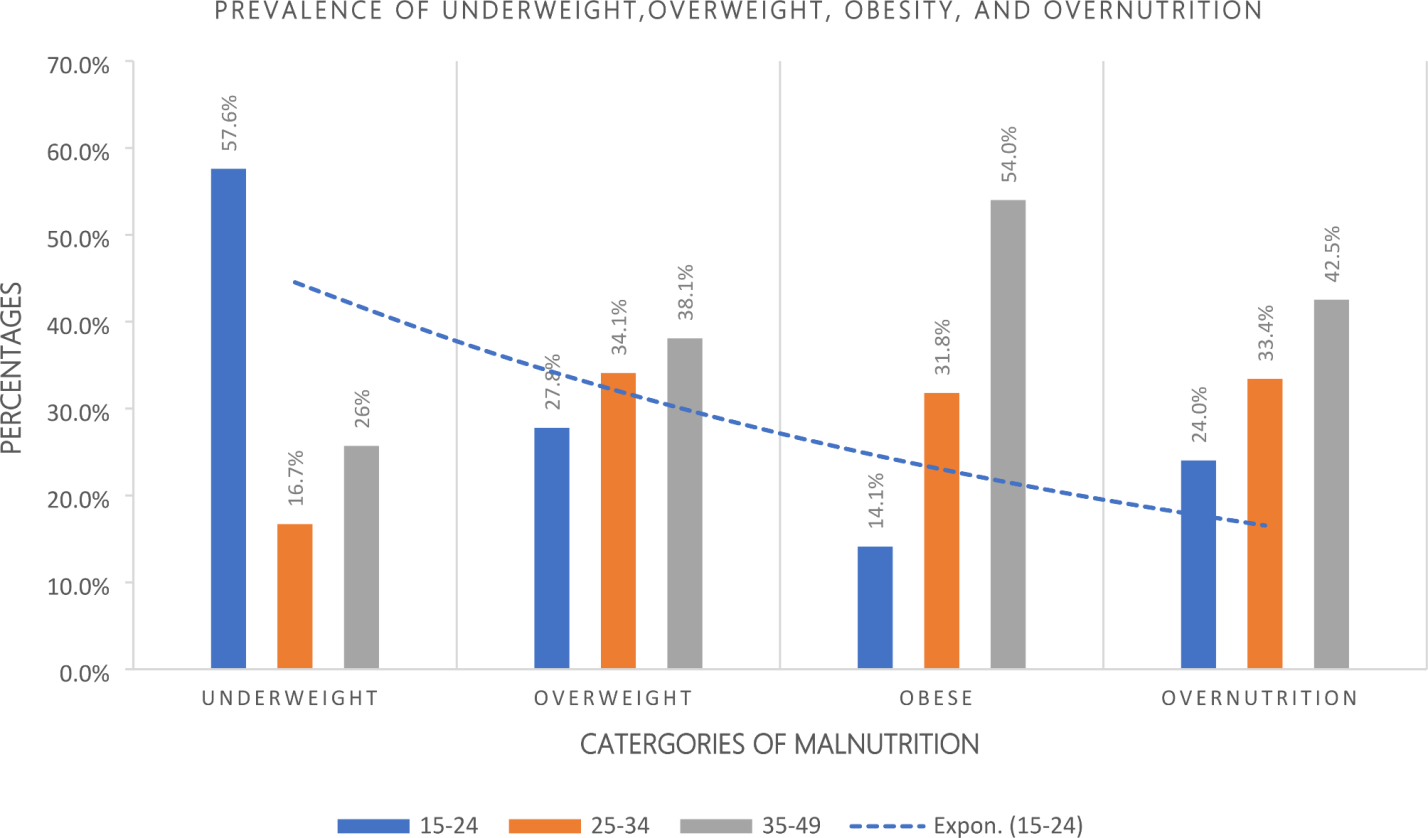
The prevalence of underweight, overweight, obesity, and overnutrition among age groups of women (15–49 years) in Sierra Leone Figure 1 shows the prevalence of underweight, overweight, obesity, and overnutrition among age groups of women (15–49 years) in Sierra Leone. Underweight women constituted 502(6.7%), overweight 1,479(19.68%), obesity, 559(7.4%), and overnourished, 2,038(27.12%). Most underweight women were in the age group 15–24 years (57.6%), overweight in 35–49 years (38.1%), obese in the 35–49-year age group (54.0%), and overnutrition in the 35–49-year age group (42.5%)

In this study, the prevalence of underweight, overweight, obesity, and overnutrition among age groups of women (15–49 years) in Sierra Leone were described. Underweight women constituted 6.7%, overweight 19.7%, obesity 7.4%, and overnutrition, 27.1%. Most underweight women were in the age group 15–24 years (57.6%), overweight in 35–49 years (38.1%), obesity in the 35–49-year age group (54.0%), and overnutrition in the 35–49-year age group (42.5%). The graph shows that overweight, obesity and overnutrition were more prevalent in the older age group of 35–49 years (Fig. 1).

## Discussion

This study is one of the first to provide evidence of a nationwide prevalence and factors associated with underweight, overweight, obesity, and overnutrition among 15-49-year-old women of reproductive age in Sierra Leone (Tables 1, 2 and 3; Fig. 1). To ensure the optimum generalizability of our findings, we used nationally representative data from the Sierra Leone Demographic Health Survey of 2019 (SLDHS-2019) [20, 25].

Specifically, this study determined the prevalence of underweight, overweight, obesity, and overnutrition among women of reproductive age (15–49 years) in Sierra Leone, with underweight at 6.7%, overweight at 19.7%, obesity at 7.4%, and overnutrition at 27.1%.

The prevalence of underweight was lower than studies conducted in other sub-Saharan African countries, for example, Kenya (9%) [29] and Tanzania (10%) [30], but similar to a study conducted in Nigeria (6.7%) [31]. This prevalence is also within the range of (5 to 20%) for women of reproductive age (15–49 years) in the African continent [31].

The observed variance in the prevalence of underweight in Kenya, Nigeria, and Tanzania were likely due to differences in characteristics of study participants, such as age and food security status [31].

In a Nigerian study by Senbanjo et al., only women aged 15–39 years from one state of Lagos were included in the survey, while the other two studies from Tanzania and Kenya included ages of 15–49 years, like ours [31]. Another study in the East African region found that Uganda, a country with the lowest food insecurity, had a lower prevalence of underweight among women of reproductive age than Tanzania and Kenya [32]. Compared to Asian countries, the Sierra Leone prevalence rate of underweight at (6.7%) is lower than Indonesia at (11.2%) [33] and Bangladesh at (30.4%) [32].

Therefore, the observed differences in the prevalence of underweight among women of reproductive age in the five countries of Uganda, Kenya, Tanzania, Nigeria, and Sierra Leone were likely due to differences in characteristics of study participants, such as the country of origin and their food security status.

The finding in the current study that the prevalence of underweight was highest among 15-24-year-olds (11.9%) and lowest among women of 25–34 years (5.6%) could be that young women have increased nutritional demands, have food insecurity, or have unhealthy feeding habits thus increasing their vulnerability to underweight.

A study by Akseer et al. showed that younger adolescent mothers (< 20 years) were more likely to be underweight than older mothers (20–49 years) in Afghanistan because of increased mother-to-child nutritional demands and increased nutritional requirements of adolescent mothers [34].

Furthermore, our study revealed that respondents of the age group (25–34 years), residing in the North of Sierra Leone, not listening to radios, and unmarried/single were significantly less likely to be underweight (Table 4). Also, the study found that parity, residency (rural versus urban), female-headed households, household size, work status, level of education, wealth indices, reading magazines, smoking cigarettes, and alcohol use were not significant correlates of underweight in this study population (Table 4).

Contrary to our findings, previous research found a significant association between lower socioeconomic status and underweight [35–37]. One possible explanation is the difference in methods used for measuring economic status, with the current study limiting it to the number of adult household members, assets, and property. In contrast, other researchers have used more composite economic indicators [35–37].

Also, findings of a double burden of malnutrition that is underweight (6.7%) and overnutrition (27.1%) in Sierra Leone, a low-income country (Table 7), are not unique but are worrisome as policymakers will need to design comprehensive public health programs that address the extremes of malnutrition. The co-existence of underweight and overnutrition has been reported in many low-income countries in the Asian Pacific region [38] and in low-to-middle-income countries, including sub-Saharan Africa [9–14]. The evidence of underweight, overweight, obesity, and overnutrition are in this study in Sierra Leone.

Many studies suggest that a rapid dietary and lifestyle transition is the leading path for the double burden of malnutrition, with an increase in overnutrition and diet-related non-communicable diseases (NCDs) [38, 39]. We, the authors, propose a need for increased efforts on policy initiatives and lifestyle changes in Sierra Leone to combat the double burden of malnutrition, which is highly prevalent in the country.

In addition, the predictors of overweight, obesity, and overnutrition among the study population include age groups of 25–34 years and 35–49 years, unmarried/single women, working-class women, women from the North and South of Sierra Leone, middle, richer, and richest wealth indices; and not listening to radios. The predictors of obesity and overnutrition were like that of overweight, with the addition of female-headed households.

The current study finding that overweight, obesity, and overnutrition were more prevalent in the older age groups is consistent with previous studies [40–42]. However, contrary to previous studies [40, 41, 43], associations between higher economic status (richer and richest wealth indices), educational level, and residing in urban areas with being overweight or obese were not statistically significant in this current study.

Of concern was that (18.7%) of young girls and women aged 15–24 was overnourished, indicating a large proportion of overweight and obese women in early adulthood. This finding means that overweight and obesity investigations following the lifecycle of women in Sierra Leone should be prioritized, as in many low-to-middle-income countries [43].

In terms of dietary behaviors, previous research found inadequate fruit and vegetable intakes [44, 45], eating occasions away from home [46], high salt intakes [47], consumption of ultra-processed foods, and saturated fats as independent predictors of obesity [48]. However, our current study being a secondary data analysis from the SLDHS-2019, we could not assess the associations between dietary behaviors such as frequency of snacking, skipping breakfast, high intake of sugary beverages, and overweight or obesity [45, 49], and overnutrition among the study population.

Unlike previous research [50–52], this study showed no associations between current tobacco and alcohol use and the prevalence of overweight, obesity, and overnutrition among the study.

### Working-class women, overweight, obesity, and overnutrition

Our study found that working-class women were less likely to be overweight but had no association with obesity and overnutrition among respondents. Previous studies from Addis Ababa, Ethiopia, reported higher figures for overweight and obesity, ranging from (26.7 to 38%) among workers in the Wonji Shewa sugar factory [53–56]. The availability of more energy-dense fast foods and exposure to sedentary life in Addis Ababa and its surroundings (compared to other urban settings) explains the high figures for overweight and obesity in Ethiopia [53–56]. The observed overweight and obesity among sugar factory workers in Ethiopia was likely because of their unfettered access to cheap and free sugar from their workplace.

Scholars, academicians, and health specialists have reported that sedentary life alone may not be the only reason for high rates of overweight and obesity as it is becoming clear that physical exercise alone does not contribute to weight loss, much as exercise is a healthy lifestyle. This finding in Sierra Leone on overnutrition at (27.12%) is lower than most studies in Ethiopia [55–58]. The Sierra Leone finding is also lower than other studies based on DHS data from Nigeria (26.7 and 36.4%) [57, 58] and seven African countries (average prevalence of 31%) [59]. It is also lower than other studies conducted in Benin (41.3%), South Africa (56.6%), Iran (61.3%), and India (75.33%) [60–63]. Therefore, this current study’s findings may differ due to disparities in dietary patterns, lifestyles, level of urbanization, and economic development in Sierra Leone.

Of particular interest in this Sierra Leone study was that it was less likely to have overweight women among the working class, contrary to findings from other African countries. We, the authors, have asked many questions about whether there is something we can learn from the results among women in Sierra Leone that can be replicated elsewhere in Africa. Could there be some dietary discipline and good dietary habits among working-class women in Sierra Leone? This question can only be answered by conducting a comprehensive qualitative study on working-class women in Sierra Leone on their dietary habits and discipline.

In addition, the current study found that the age of women, marital status, wealth indices, working-class women, female-headed households, and residence in the North and South of Sierra Leone were significantly associated with overweight, obesity, and overnutrition among women of reproductive age. Consistent with other studies, this study’s results demonstrate that overweight and obesity are higher among older women [64–68]. A decrease in levels of physical activities and higher intakes of energy-dense foods as the age of women advances is suggested as a possible explanation [69].

In contrast, being overweight, obese, and overnourished was less likely among women in the middle, richer, and richest wealth indices in Sierra Leone women. This finding is inconsistent with studies from Addis Ababa and Wonji Shewa sugar factory [51, 64, 65] and elsewhere [61–63, 66, 67].

In developing countries, wealthier women are more likely to consume more energy-dense foods and follow a sedentary lifestyle; hence, they are more likely to be overweight, obese, and overnourished [61–67]. However, this was not the case in Sierra Leone, where overweight, obesity, and overnutrition were less likely among working-class women of reproductive age (15–49 years). As authors, we believe there is a need to explore these unique findings among women in Sierra Leone in future studies.

Furthermore, previous studies showed that the prevalence of overweight, obesity, and overnutrition was significantly higher among working women with higher educational levels [67, 68]. However, a higher educational level was not statistically significant in our study population except for the crude odds ratios for respondents with a primary level of education and obesity.

Our current study finding on the level of education and association with obesity contrasts with other studies where a higher educational level is associated with obesity [67–69]. This finding on obesity in different settings may be a result of changes in lifestyles as disposal income rises; these classes of women tend to go for processed carb diets and more sugary drinks, including drinking tea three to four times a day with bread with a shift from manual labor to more sedentary occupations and the related decline in physical activities.

In contrast to other studies, the unmarried/single women in the current study were independent predictors of overweight, obesity, and overnutrition [70–72]. Previously, married women were more likely to have higher parity, resulting in adopting a more sedentary lifestyle and eating high-energy foods, usually offered to women during the postpartum period, thus becoming overweight or obese. On the contrary, we found that unmarried/single Sierra Leone women were more likely to be overweight, obese, and overnourished.

On this finding, we, the authors, suggest that perhaps many unmarried/single women in Sierra Leone lead a more sedentary lifestyle, have a higher energy-rich diet, and are from the northern region, and this may, in part, explain the associations between the unmarried/single with overweight, obesity, and overnutrition among women in the reproductive age in this country. These authors cautiously recommend further studies to determine why single/unmarried women were more likely to be overweight, obese, and overnourished in Sierra Leone compared to findings from other countries in Sub-Saharan Africa.

### Female-headed households, wealth indices, overweight, obesity, and overnutrition

Our current study found that better wealth indices and female-headed households were less likely to be associated with overweight, obesity, and overnutrition among women of reproductive age (15–49 years) in Sierra Leone.

Although there are cross-country differences, the number of populations living in female-headed households and households headed by women has risen over the years [73]. Current data show that the probability of a woman aged fifteen or older in households, controlling for her age, has been increasing since the early 1990s in all regions and across the entire age distribution in Africa [73].

Using a complete series of DHSs fielded in Africa over the last 25 years and covering 89% of Africa’s population, recent research has investigated Africa-wide changes in the prevalence of female-headed households [73]. The result suggests that economic growth brings more female headship, presumably due partly to lower work-related migration by men but associated with a growing local economy [73].

The seeming paradox that female headship is rising during a period of growth is that other things are also changing across Africa [73]. Changes in the demographic and population characteristics, social norms, women’s education, and the family’s nature are encouraging female household headships in the African continent [73].

Current reports show that an extra year of schooling produces a three-percentage increase in shares of the population living in female-headed households [73].

In addition, on average, a one-year rise in women’s age at first marriage produces a 2.5%-point increase in the share of the population living in female-headed households, an effect almost as strong as an extra year of schooling [73]. Life expectancy’s positive impact equals a 0.5%-point boost per additional year among women, presumably reflecting the natural survival advantage of women with higher overall life expectancy and the resulting incidence of widow-headed households [73].

Furthermore, conflicts, wars, and HIV and AIDs in many communities in the African continent have raised many countries’ share of the population in female-headed households [73]. Thus, female-headed households’ prevalence has been rising while poverty has been falling in Africa [73]. Past literature has generally been suggestive that female-headed households tend to be poorer. Still, the critical question is whether this occurrence implies that recent improvements in living standards have left behind female-headed households [73].

On the contrary, female-headed households are a diverse group of people [73]. Some, such as married women with non-resident husbands (polygynous or migrant) or educated women who may choose, and socially and economically afford not to be married or remarry, can be expected to be relatively well-off [73]. Other scenarios, such as wars or AIDS widows, separated or abandoned women, and single mothers who have not chosen headship but have no options, are frequently found to head disadvantaged households [73].

So, the finding in our current study that it was less likely to have overweight, obese, and overnourished women among better wealth quintiles and female-headed households attracts interest since previous studies appear to inform that better wealth quintiles were associated with obesity, overweight, and overnutrition.

In addition, poverty declined for both household groups, but in most African countries, it fell faster for female-headed households (FHHs) than for MHHs [73]. This finding is also factual when one allows for diversity among FHHs; for example, comparing households with widow and non-widowed heads, married heads with and without a male adult household member, and the same for non-married heads and the finding that poverty is falling faster for FHHs is robust for testing sensitivity in allowing for the generally smaller size of FHHs and economies of scale in consumption, which does not alter these key findings [73].

In addition, the standard of living of the various types of FHHs followed different paths across countries and periods, with no one type consistently outperforming the others, yet at least one of the types of FHH usually surpassed male-headed households (MHHs) [73]. Furthermore, there needs to be a more discernible pattern across African countries; notably, one category of FHH may do well in one country or a period while another does best elsewhere [73].

Remarkably, poverty has fallen more rapidly in FHHs in the African continent. A decomposition in changes in poverty indicates that rather than put a break on poverty reduction, FHHs are contributing appreciably to the decline of poverty despite their smaller share in the population [73].

Nevertheless, the big question is, why has poverty fallen faster for FHHs? There are different explanations, but perhaps poor FHHs faced a relatively high economic return to the new opportunities unleashed by growth, or maybe they have benefited disproportionately from the expansion of social protection in the region, or perhaps the group of people living in FHHs is fundamentally changing over time [73].

This finding in our current study means that a superficial examination of FHHs and better wealth indices observed from this study may not support any of these explanations. Still, this newly stylized fact about poverty in Africa warrants a closer look in the future.

### A double burden of malnutrition (DBM) in Africa

We, the authors, argue that among the drivers of the DBM, poverty-related factors, such as food insecurity and infectious diseases, persistent droughts, floods, gender prejudices, and protracted humanitarian crises, continue to mark the face of Africa [74]. For overweight and obesity, cultural expectations and the early onset of puberty predispose girls to high adiposity and lifestyles [75]. Cultural perceptions of sizeable female body size also drive DBM, as being overweight is considered a sign of wealth, achievement, and marital harmony [75]. This cultural aspect and reduced physical activity could explain why obesity is consistently higher in women than men in Africa [75]. Meanwhile, consumption of processed foods is increasing at the expense of fresh and minimally processed foods among the African population [75, 76]. The commercialization of food production, processing, and distribution is correlated with decreasing smallholder farming, dietary diversity, and increasing household dependence on purchased foods, resulting in diets of low nutritional quality, energy-dense, and high in sugars, salt, and fats [76]. The underlying causes of the DBM may vary by subregion, but the increasing consumption of cheap, processed foods [77] and reduced physical activity are among the key drivers of a DBM.

In summary, the prevalence of overweight and obesity exceeds underweight in most women of reproductive age (15–49 years) and are risk factors for cardiovascular diseases in developing countries [78–80]. Findings from Sierra Leone show that it is no exception to the growing prevalence of DBM among women of reproductive age.

Of particular interest in the Sierra Leone case, the number of adolescents underweight, overweight, obese, and overnutrition is relatively high. Suppose this prevalence of the different malnutrition categories is not arrested in Sierra Leone, we, the authors, predict that we shall begin to observe higher incidences and prevalence of NCDs, poor obstetric outcomes, and disadvantaged offspring in the coming years.

### Strengths and limitations of this study

First, the strengths of this study were the use of nationally and sub-nationally representative data and considerations of complex sampling methods. Second, the use of validated questionnaires and calibrated tools that were used for data collection provides credence for these results. Third, the sampling method of respondents was a two-stage stratified probability sampling method where the selection of respondents was robust and representative of the population.

However, four limitations should be considered while interpreting the results of this study:

1. The study’s cross-sectional nature does not allow for establishing causality of associations.
2. Significant predictors for the outcome variables, such as physical activity and total energy intake (nutritional history), food availability, and types consumed, were absent due to the secondary nature of the data available.
3. Likewise, there was no data on central obesity since the survey did not collect data on abdominal and waist-to-hip circumferences.
4. Apart from physical and biomedical measures of the self-reported questionnaire, this data may have suffered from social desirability biases.
5. Although body mass index (BMI) is widely used as a first-line screening biomarker for nutritional status assessment, the advantages of BMI are its simplicity, low cost, and non-invasiveness [81, 82]. However, this biomarker has several limitations, which lead to low sensitivity in the diagnosis of both malnutrition and obesity; for example, more than half of the people with a high percentage of body fat (e.g., > 30%) are diagnosed as being in the BMI range for an average weight [81, 82]. The shortcomings of BMI as a biomarker of malnutrition depend on (i) the slow effect of decreased food intake on its value and (ii) its weak correlation with biochemical and immunological parameters of malnutrition [81, 82].

Although the limitations of BMI as a biomarker of obesity are related to (i) an inability to distinguish between fat and fat-free (lean) body mass, (ii) a failure to determine fat distribution, (ii) a dependence on the accuracy of reported or measured height; and (iii) the influence of age, gender, and comorbidities on the accuracy of the cut-offs used in the diagnosis of obesity [81, 82].

Nevertheless, BMI correlates with (i) central body fat distribution, (ii) laboratory biomarkers of metabolic (e.g., blood glucose, lipids, uric acid), inflammatory factors (e.g., c-reactive protein, interleukin-6, and tumor necrosis factor-alpha), and endothelial (e.g., VEGF and ICAM) abnormalities [81, 82].

In addition, BMI is also useful as (iii) a risk factor (biomarker) in the development of many health conditions, such as diabetes mellitus, hypertension, infectious disease, and psoriasis; (iv) as a prognostic factor for all-cause and cardiovascular mortality, in-hospital all-cause mortality, surgery complications and outcomes, hospital-acquired (nosocomial) infections, length of in-hospital stay, and risk of readmission; as well as (v) a biomarker for monitoring the clinical and metabolic effects of interventions on weight reduction, including bariatric surgery [81, 82].

### Generalizability of the results

Results of this study can be generalized to low-resource settings, particularly in low-to-middle-income countries.

## Conclusion

The prevalence of all malnutrition categories among women of reproductive age (15–49 years) in Sierra Leone was high, affirming a double burden of malnutrition in this study population. Underweight was more likely among the 25–34-year age group than 15–24-year. The predictors of overweight, obesity, and overnutrition were unmarried/single women, women from the North, and not listening to the radio. There is an urgent need for policymakers in Sierra Leone to design comprehensive educational programs to sensitize, engage, and mobilize women in the reproductive age group on healthy lifestyles and the dangers of being underweight or overnourished.

## Data Availability

All datasets supporting this article’s conclusion are within the paper and are accessible by a reasonable request to the corresponding author.

## Abbreviations

aOR: Adjusted Odds Ratios
CI: Confidence Intervals
DBM: Double Burden of Malnutrition
BMI: Body Mass Index
DHS: Demographic Health Survey
FHHs: Female-Headed Households
IRB: Institutional Review Board
NCDs: Noncommunicable Diseases
OWOB: Overweight and Obesity
SL: Sierra Leone
SLESRC: Sierra Leone Ethics and Scientific Review Committee
WHO: World Health Organization
